# An Exploration of Mental Health Discussions in Live Streaming Gaming Communities

**DOI:** 10.3389/fpsyg.2021.575653

**Published:** 2021-03-16

**Authors:** Reesha Gandhi, Christine L. Cook, Nina LaMastra, Jirassaya Uttarapong, Donghee Yvette Wohn

**Affiliations:** Social Interaction Lab, Department of Informatics, New Jersey Institute of Technology, Newark, NJ, United States

**Keywords:** live streaming, Twitch, interviews, digital games, online communities, self-disclosure, online discussions, mental health

## Abstract

Live streaming is a unique form of media that creates a direct line of interaction between streamers and viewers. While previous research has explored the social motivations of those who stream and watch streams in the gaming community, there is a lack of research that investigates intimate self-disclosure in this context, such as discussing sensitive topics like mental health on platforms such as Twitch.tv. This study aims to explore discussions about mental health in gaming live streams to better understand how people perceive discussions of mental health in this new media context. The context of live streaming is particularly interesting as it facilitates social interactions that are masspersonal in nature: the streamer broadcasts to a larger, mostly unknown audience, but can also interact in a personal way with viewers. In this study, we interviewed Twitch viewers about the streamers they view, how and to what extent they discuss mental health on their channels in relation to gaming, how other viewers reacted to these discussions, and what they think about live streams, gaming-focused or otherwise, as a medium for mental health discussions. Through these interviews, our team was able to establish a baseline of user perception of mental health in gaming communities on Twitch that extends our understanding of how social media and live streaming can be used for mental health conversations. Our first research question unraveled that mental health discussions happen in a variety of ways on Twitch, including during gaming streams, Just Chatting talks, and through the stream chat. Our second research question showed that streamers handle mental health conversations on their channels in a variety of ways. These depend on how they have built their channel, which subsequently impacts how viewers perceive mental health. Lastly, we learned that viewers’ reactions to mental health discussions depend on their motivations for watching the stream such as learning about the game, being entertained, and more. We found that more discussions about mental health on Twitch led to some viewers being more cautious when talking about mental health to show understanding.

## Introduction

In the entertainment industry, live streaming video games has become increasingly popular and lucrative (Nascimento et al., 2014), attracting people of all types in the gaming community (Kaytoue et al., 2012). Twitch.tv has become especially popular among gamers for allowing people to stream their own video game content, particularly if they are professional gamers, as well as watch others stream (Nascimento et al., 2014). Over time, Twitch became a popular streaming service (Twitch, 2020) for both casual and professional gamers to create a community and become microcelebrites (Anderson, 2017), who are people that promote their identities as a brand through online mediums (Senft, 2013). Through such a community, the audience is more inclined to interact with their favorite internet personalities and watch skilled players win their favorite games (Sjöblom and Hamari, 2017).

Previous research has found that people enjoy watching video game live streams more if they believe they will benefit from the stream in some capacity, whether this is by being entertained (Hilvert-Bruce et al., 2018), releasing tension (Sjöblom and Hamari, 2017), acquiring information (Sjöblom and Hamari, 2017), or gaining a sense of community (Chen and Lin, 2018). It is, however, the sense of community formed through chat on and outside of Twitch.tv that primarily keeps viewers coming back to their favorite streams (Sjöblom and Hamari, 2017). This sense of community also directly impacts how people of influence affect those in their community, especially when it comes to their health-related behaviors (Hoffman and Tan, 2015), as shown by research on other types of online communities (Giles, 2002; Hoffner, 2019). Because microcelebrities have a powerful influence over their followers, those who disclose their mental health tend to have a large influence in reducing mental health stigma as well (Hoffner, 2019).

There have been many studies detailing how mental health is perceived and communicated on various social media platforms such as Facebook, Twitter, and Reddit, but very few studies explore the issue in relation to live-streams (Egan et al., 2013; Berry et al., 2017; Park and Conway, 2018). Twitch’s live streaming platform allows its streamers to cultivate a community around their content because of Twitch’s main focus on gaming, as opposed to other platforms such as YouTube that are more general (Pires and Simon, 2015). This study is among the earliest to analyze how mental health is discussed in live streaming gaming communities, specifically on Twitch. It is also among the earliest to look at game watching’s mental health impact, as most of the literature surrounding gaming and mental health emphasizes the impact of playing games on mental health (e.g., Kaye et al., 2017; Carras et al., 2018).

This study aims to uncover how the gaming live stream community perceives and discusses mental health. The first goal of this study is to determine how mental health is discussed in game streams. While many studies have discussed gaming disorder and addiction to gaming (Kuss and Griffiths, 2012; Jones et al., 2014; Aarseth et al., 2017), our goal is to look at how the communities surrounding gaming and the Twitch.tv platform itself can either support or inhibit viewers’ discussions around mental health. The second goal of this study is to explore how discussions about mental health affect the gaming community on Twitch. Through analyzing viewer’s perceptions of mental health discussions in the gaming community and how different parts of the gaming community react to mental health, this study will contribute to unveiling the significance of mental health in Twitch gaming communities.

## Literature Review

### Live Streaming and Twitch.tv

The live streaming of games, or “game-streaming” as Lin et al. (2019) call it, is a popular pastime, with 3.8 million people around the world streaming video games in the month of February 2020 on Twitch.tv alone (Iqbal, 2020; TwitchStats, 2020). The games being streamed vary considerably, and both digital and tabletop games are represented (Johnson, 2019). The most important components to a game stream are that there is a game being played and an option for someone else to watch, as viewership can vary considerably depending on the game and the channel (Wohn, 2019). Still the largest live streaming service around (Stephen, 2020), gamers can use Twitch to watch and learn how professional esports stars play (e.g., Burroughs and Rama, 2015; Johnson and Woodcock, 2017; Brown et al., 2018), join an entertaining community of like-minded people (e.g., Diwanji et al., 2020), indulge in nostalgia (e.g., Gandolfi, 2016), or even direct their favorite personality in crowdsourced live streams (e.g., Zhang and Liu, 2015). For some lucky few, live streaming platforms such as Twitch can even be a primary source of income (Carras et al., 2018; Velez et al., 2018).

### Social Motivations of Watching Live Streams

While previous live streaming research indicates entertainment and knowledge to be the most common motivations for watching live streams, some studies stress the importance of social interactions through participating in live streams (Hamilton et al., 2014; Chen and Lin, 2018; Gros et al., 2017). Many viewers watch streams to converse with likeminded people, and prior literature has suggested that the communities that form around streamers may help alleviate viewers’ feelings of self-consciousness and loneliness (Hamilton et al., 2014; Hamari and Sjöblom, 2017; Hilvert-Bruce et al., 2018). Many streamers recognize the importance of social interactions in their communities, and therefore make an effort to be responsive to their followers by having discussions, reading out subscription messages on Twitch, and using polls (Hamilton et al., 2014; Chen and Lin, 2018). As noted by Chen and Lin (2018), how a viewer views a streamer—trustworthy, charming, or attractive, for example—significantly relates to how comfortable they feel interacting with others on stream. A total of 70% of Twitch streamers play a variety of games not specific to a genre; as such, some viewers prefer watching streamers not only for the content of the stream, but for the personality of the streamer themselves (Deng et al., 2015). This suggests that a streamer’s personality has a direct impact on how viewers act online. Celebrities and microcelebrities can thus significantly influence one’s physical and mental health-related behaviors.

### Mental Health Stigma

Mental health stigma is defined as the negative reactions and “prejudicial beliefs and actions toward people who are viewed as ‘different’ or ‘lacking”’ (Aguiniga et al., 2016, p. 429). Previous mental health literature suggests that the public’s mental health literacy should be raised (Jorm et al., 1997; Kelly et al., 2007), indicating that some of the public does not fully comprehend mental health as a concept. One study reported its participants viewing the cause behind social stigma as a lack of understanding from the general public when it comes to mental health (Vidourek and Burbage, 2019). This lack of understanding leads to greater social stigma, as the populous cannot identify with or relate to those living with mental health disorders (Vidourek and Burbage, 2019). As social stigma rises, so does its effect on the individual; it intensifies self-stigma by creating feelings of guilt and inadequacy, causing a negative impact on one’s self-esteem.

When public figures discuss their personal health issues, there is an increase in internet searches on mental health issues, subsequently spreading awareness of these issues among their followers (Lee et al., 2020). Robinson (2019) also found that discussions surrounding mental health on Twitch.tv were becoming less toxic and more frequent and positive, with one microcelebrity hosting a “Twitch Therapy” stream among their usual gaming streams. Robinson (2019) then suggests that this increase in discussion may relate to a more positive mindset about mental health. Of critical importance to this study is the fact that many of these microcelebrities talk about mental health while playing video games to develop meaningful relationships with viewers (Carras et al., 2018).

This study aims to uncover how the gaming live stream community perceives and discusses mental health. Our goal is to focus on how the communities surrounding gaming and the Twitch.tv platform can either support or inhibit viewers’ discussions around mental health. This study will examine the following research questions: (1) How do mental health discussions occur in game streams? (2) How do streamers facilitate discussions about mental health in their community? and (3) What is the viewer reaction to mental health discussions in the gaming community? Through analyzing viewers’ perceptions of mental health discussions that they have witnessed on gaming live streams, this study provides an understanding for future studies surrounding gaming communities on Twitch.

## Materials and Methods

### Participants

While recruiting, we targeted Twitch viewers who have witnessed mental health discussions during live streams. Our recruitment process began with reaching out to participants from a prior study that surveyed attendees of TwitchCon^1^ 2019. Seven out of the 229 people who were contacted responded to our recruitment efforts and were interviewed.

The rest of our participants were recruited using recruitment posts on Reddit, Twitter, Facebook, Twitch, and gaming-focused Discord servers. Additionally, a few participants (*n* = 4) were recruited from our personal networks. Our recruitment message told participants the study will have them discuss their experiences with mental health conversations on Twitch. Each participant signed a virtual consent form prior to being interviewed. As a result, we conducted a total of 25 interviews primarily using Discord, with the exception of one interview conducted via phone call. The data used in this study came solely from these interviews. Table 1 contains further information about each participant, including demographic information, whether they are a streamer, a moderator^2^, or both on Twitch, and how we recruited them. Participants ranged in age from 18 to 39 years old (*M* = 25.36, SD = 5.484), and the majority of these were male (16; eight female; and one non-binary) and Caucasian (18; two Asian; and five Hispanic).

**TABLE 1:** Descriptions of participants’ demographics and recruitment information.

Number	Age	Race*	Gender	Streamer	Moderator	Recruitment method
1	20	Caucasian	Female	Yes	No	Personal contact
2	24	Hispanic	Male	Yes	Yes	TwitchCon 2019 attendee
3	36	Caucasian	Male	No	Yes	TwitchCon 2019 attendee
4	34	Caucasian	Female	No	Yes	TwitchCon 2019 attendee
5	20	Caucasian	Female	Yes	No	Personal contact
6	24	Hispanic	Female	No	No	TwitchCon 2019 attendee
7	22	Caucasian	Female	Yes	No	TwitchCon 2019 attendee
8	33	Caucasian	Male	No	No	TwitchCon 2019 attendee
9	27	Caucasian	Male	Yes	No	Reddit
10	23	Hispanic	Female	Yes	No	TwitchCon 2019 attendee
11	19	Caucasian	Male	No	No	Reddit
12	28	Caucasian	Female	Yes	Yes	Twitter
13	21	Asian	Male	Yes	No	Reddit
14	39	Caucasian	Male	Yes	No	Twitter
15	23	Caucasian	Male	Yes	No	Twitter
16	23	Asian	Male	No	No	Discord
17	28	Caucasian	Male	No	Yes	Personal contact
18	18	Caucasian	Male	Yes	Yes	Discord
19	21	Caucasian	Male	No	No	Discord
20	22	Hispanic	Male	No	No	Discord
21	26	Caucasian	Male	No	No	Twitch
22	29	Caucasian	Female	Yes	Yes	Reddit
23	21	Hispanic	Male	Yes	No	Personal contact
24	28	Caucasian	Non-binary	No	Yes	Twitch
25	25	Caucasian	Male	Yes	No	Personal contact

*Self-reported.

### Procedure

These interviews lasted an average of 59 min (SD = 25.459), ranging from 32 min to 2 h long. The participants received a payment of $20 USD, with the exception of two participants who declined payment. The interview protocol was semi-structured, beginning with questions aimed to understand what type of viewer the participant was, the types of communities on Twitch they engage with, and whether the participant is also a streamer, a moderator, or both (see Supplementary Material for full interview protocol). Participants were then asked to describe their general impressions of mental health conversations on Twitch. This includes how frequently they observed conversations, how they were initiated, and how they and other viewers reacted. We then asked participants to describe their favorite gaming streamer, their relationship to that streamer, specific mental health conversations they witnessed on that streamer’s platform, if the participant had learned anything related to mental health, and if the streamer promoted any charities. Details we asked for included specific disorders mentioned, whether the conversations were mainly personal experiences, and how viewers reacted to these discussions. Interviews closed with questions regarding general opinions about mental health outside of Twitch and the participant’s personal experiences with mental health.

### Analysis

Interviews were audio recorded and then transcribed via an online transcription service, with at least one author reviewing each transcription. We then used the transcribed interviews to explore our research questions and the types of communities each participant was active in. Next, we identified answers to interview questions closely associated with our research questions and recorded these answers onto a color-coded spreadsheet. Each row in the spreadsheet represented a specific participant, while each column represented a specific question. Two columns were also dedicated to miscellaneous quotes that may be relevant to one or more research questions.

For each research question, we independently distilled each quote into codes based on its main idea using a meta matrix. As codes were developed, we created a codebook that had an explanation of each of the codes that were generated. The team met periodically to discuss the codes as they emerged and iteratively revised the codes and codebook as the data analysis proceeded. After at least two authors had coded the participants’ responses, all authors reviewed the codes and data together to ensure that we were in agreement. We then discussed what themes were present in each research question using a grounded theory approach (Strauss, 1987). We had 99 codes, compiled them into 53 themes, and then merged into 13, for all three research questions (see Tables 2–4).

**TABLE 2:** Coding breakdown for RQ1: how mental health discussions occur during game streams.

Theme	Sub-theme	Codes
Streamer-initiated	Off-stream/via social media	Streamer-initiated, only social media, off-stream, Twitter, Instagram
	Scheduled vs. spontaneous	Streamer-initiated, scheduled time, spontaneous, during game, unaffected, affected, content dependent, routine, Runescape, downtime, stop game, streamer mentality, just chatting, streamer stories, on stream, in between games, streamer dependent, League of Legends, support from streamer
	Special guests	Streamer-initiated, Destiny, advocating for professional help
	Charity streams	Streamer-initiated, sub/donation
Chat-initiated	Personal stories being shared	Chat-initiated, sympathizing/empathizing, support from community
	Cries for help	Chat-initiated, streamer uncertainty, support from community
	Donations/subscriptions prompting the conversation	Chat-initiated, sub/donation, content dependent, streamer dependent, dismissive, support from streamer
		

**TABLE 3:** Coding breakdown for RQ2: how streamers facilitate mental health discussions.

Theme	Codes
Spreading awareness	Community sharing personal stories, spreading awareness, fundraising, streamer support, directing to resources
Connecting with viewers	Moderated chat, strong community social norms, positive reactions, viewer/community support, not purpose of Twitch, streamer empathizing, infrequent discussion, frequent discussion, becoming more common, game-dependent, positivity advocate
Moderating mental health discussions	Trolls, self-moderating chat, dropped viewer count, streamer persona, ignore negative chat, streamer-dependent

**TABLE 4:** Coding breakdown for RQ3: viewer reaction to mental health in the gaming community.

Theme	Codes
Emotional response	Joy, indifferent, discomfort, comfort, well-moderated chat, negative emotions, encouraging, making friends, empathy, engaging with streamer, mood-dependent
Offering support	Sympathizing, sharing personal stories, offering support, offering advice, participant empathizes, viewers empathize
Respect for streamers	Respect for streamer, increased focus, entertainment vs. personality, dropped viewer count, in moderation
Spreading awareness	Stigma reduction, spreading awareness, acceptance, careful response, catharsis vs. attention, humor, discussion-provoking

## Results

### How Mental Health Discussions Occur During Game Streams (RQ1)

Our first research question asked how mental health discussions occur in game streams. With a large variety of content on Twitch spread throughout vastly different streamers and communities, mental health discussions emerge in many ways. To delve into how these discussions happen, we look into who initiates these discussions, how they initiate them, and what happens in reaction to them.

#### Streamer Initiated

##### Off-stream/via social media

While our study’s main focus is on mental health conversations during live streams, it is important to note that in association with many live streams, some off-stream conversations occurred. Participants noted how streamers use other social media platforms, such as Twitter and Instagram to communicate to their followers, sometimes going into more detail off stream than on stream. Some streamers, such as the streamer P1 (female, 20, Caucasian) references, only discuss mental health topics off stream. Furthermore, P19 (male, 21, Caucasian) details how the streamer they watch, RTGameCrowd, makes bi-weekly videos about updates on his mental health and discusses the topic more on his YouTube than on Twitch.

##### Scheduled vs. spontaneous

Mental health conversations were found to occur in both spontaneous manners as well as ones scheduled by the streamer. P21 (male, 26, Caucasian) described how, since the streamer he watches is a coach, analyzing the mentality of players is already part of his routine. P21 also describes how his streamer of choice has coaching materials easily available while streaming.

While all participants reported witnessing mental health conversations during live streams, 14 participants reported these conversations occurring during gameplay. P17 (male, 28, Caucasian) said they have seen a streamer initiates a mental health discussion spontaneously during a game that may involve intense gameplay but has fewer intensive moments, and how moments like those prompt mental health discussions: “There was nothing really in the game that happened around it… what she was doing in Runescape was more of a solo grind.”

Participants reported that gameplay was almost equally affected or unaffected depending on the intensity of the game. P19 expressed that streamer RTGameCrowd’s gameplay is unaffected as the games they play are less intensive “small indie games.” P15 (male, 23, Caucasian) spoke to this point similarly, stating:

I think that you’re more likely to hear somebody comfortably talking about their mental health during an Animal Crossing or a Stardew Valley stream or like a Pokemon stream than you are during Call of Duty … I don’t think while gunshots are going off that people are going to be like, “Yeah, that’s why my parents left me with trauma.”

Participants also detailed occurrences of gameplay being affected. P9 (male, 27, Caucasian) said, “Whenever you hear that somebody is depressed or going through issues… in [the] worst case they’re like, ‘I’m feeling suicidal.’ It’s like, how can you continue to play your game?” P9 detailed an occurrence in which a viewer donated their “last five bucks” to DrLupo and told the streamer they wanted to “end it all”; DrLupo stopped the stream to directly talk to the viewer, direct them to the suicide hotline phone number, and have a discussion on mental health.

While P9 provided an example of gameplay stopping for the sake of a viewer’s mental health, streamers also stop the game in regard of their own mental health. P10 (female, 27, Hispanic) went into detail about a streamer she views who cut ties with another streamer in her demographic community. When the streamer decided to mend the relationship, she received backlash from chat, at which point she broke down and cried and told the chat how it hurt that her chat was too involved with their lives. This “impacted her game play because… she went AFK [away from keyboard]. She wasn’t concentrating on the game play anymore; her thoughts were elsewhere” (P10).

A streamer’s mood can create a spontaneous conversation related to the streamer’s mental health, regardless of whether viewers opt to provoke or sympathize with them. As prior examples depict scenarios of gameplay being affected to the point of the streamer needing to stop playing, less extreme cases lead streamers to simply prefer to partake in these discussions in-between games or to have them in exclusively the “Just Chatting” category.

The data show that streamers who have mental health discussions occurring between games or in Just Chatting are a result of the streamer’s preference as well as gaming situations. P22 (female, 29, Caucasian) provided one example of how the streamer would sometimes stop in-between player vs. player (PvP) matches to focus on the chat and partake in these discussions. P9 mentioned how they observed streamers delving into mental health topics during Just Chatting streams or in-between games because they should not be “in the middle of an Apex battle.” These examples show how streamers prefer to pause during mental health discussions because PvP games can be too intense. Connected to this idea, P23 (male, 21, Hispanic) said:

It’s some type of mental load that you have to take on to have to create a meaningful response to someone’s situation while at the same time you’re reacting to some high paced graphics that are on your screen… It does impact the game play a bit, which is why there tends to be a switch from the game to Just Chatting.

Furthermore, game specific content can be the catalyst of a spontaneous mental health discussion. P2 (male, 24, Hispanic) mentioned how the game Hellblade: Senua’s Sacrifice “talks about depression and suicide and those archetypes and how redemption works… [I] believe they even did research into schizophrenia.” Though the participants did not discuss specific ones, they mentioned that there were other games on Steam^3^ that cover mental health.

##### Special guests

Participants noted that certain streamers go out of their way to initiate mental health conversations on their streams by occasionally having special guests to help facilitate these discussions. Participants described these special guests as mental health-centric internet personalities who are trained psychologists. P12 (female, 28, Caucasian) paraphrased how drMickLive, a trained counselor, would help initiate and assist with mental health conversations during Destiny^4^ streams. P6 (female, 24, Hispanic) also shared viewing psychiatrists being special guests during Destiny streams, as well as other gaming and just chatting streams. Furthermore, P12 being a streamer themselves described featuring a mental health-centric special guest as, “killing two birds with one stone,” as this way the main streamer can focus on the game at hand, while the special guest focuses on the chat and the discussion. With this tactic, the gaming does not need to be interrupted, but the mental health discussion can proceed smoothly.

##### Charity Streams

Another avenue of contributing to mental health causes and raising awareness is charity streaming. Most participants have witnessed streamers charity stream for mental health causes but did not donate to them personally. P9 said that he only donates if a streamer is very close to a goal, but regardless of whether they donate or not, witnessing the stream is educational to both them and the other viewers.

P12, having both donated and fundraised for mental health causes, explained how certain streamers encourage viewers to donate:

The best is always when they have a good spiel about the charity and specifically about the campaign that they’re running at the moment… So when they have… [something] very tangible like, “this is how this is going to help these children” … To Write Love On Her Arms said that like $5 pays for 1 h of teleconference to do an assessment of what kind of mental health assistance someone needs.

P12 also provided examples of incentives she has seen streamers give during charity streams, such as drawing a heart on their face if a viewer donates $5, or deleting a save file when certain donation milestones are met.

Furthermore, certain charities are more well-known than others. P18 (male, 18, Caucasian) brought up how Make-A-Wish and similar charities are common knowledge, but how Comic Relief, “a mental health association that deals with people in foster homes” is lesser known. Streamers using their platforms to run charity streams not only educate viewers on the topic of mental health, but can help bring to light lesser known causes.

#### Chat-Initiated

##### Personal stories being shared

Our data show that mental health discussions are initiated by both the chat and the streamer. Within the category of chat-initiated mental health discussions, several participants mentioned that mental health discussions come up when a viewer shares a particularly heart-wrenching personal story:

There was a community member that spoke up about the fact that their mother had passed away last week and that they felt alone because this was the only family they had. And … they didn’t know what to do. They didn’t want to be without their parent, and they were afraid of. self-harming … it’s a definite, horrible thing to have to go through, but they’re not alone in feeling how they do (P3, male, 36, Caucasian).

From there, viewers often join in with sympathizing, empathizing, or encouraging messages directed at this person who is sharing. This can be within the chat portion of the stream, through direct messages, or in other social media. However, this initial sharing can also be a catalyst for the reciprocal sharing of personal stories within chat. This ends up as a community bonding experience that can create strong emotional ties between viewers. In this way, the chat develops a common ground and establishes the stream as a safe space in which these kinds of discussions can occur.

##### Cries for help

Sometimes, viewers make out-of-the-blue statements like “I’m suicidal” or “I’m feeling down” (P9, male, 27, Caucasian; P23, male, 21, Hispanic) leading to the legitimacy of these statements to come into question, as noted by P22 (female, 29, Caucasian):

Sometimes there are posts… formatted as a cringey viewer, a viewer that might fake it, or a streamer that might fake it. When it comes [to] mental health, it’s about, does this person fake it likely and how do I address this with this person? Or if I am really uncomfortable, I am not certain if they’re faking it, how can I deal with this? Can I report it?

P2 (male, 24, Hispanic) made a similar observation, stating that “they’re [the viewer in question] just making a scene in the chat and usually that will upset people.” According to P3 though, these attention-seeking viewers are “probably the exception more than the rule,” and P14 (male, 39, Caucasian) emphasizes the fact that these pleas are “just trying to get attention.” Streamers and moderators use their own subjective judgment when determining whether a comment is genuine or from those seeking attention. The general consensus seems to be that the majority of viewers making mental health pleas perceived as attention-grabbing tactics get moderated out. Thus, the vast majority of chat-initiated mental health discussions end in community bonding and mutual sharing of experience rather than additional trauma for the viewer(s) or streamer who is/are expressing their struggles.

#### Donations/Subscriptions Prompting the Conversation

Viewers may also donate or subscribe to the streamer, which in some channels posts a message attached to the donation or subscription directly onto the stream. P18 (male, 18, Caucasian) observed that “… the people who would be willing to talk about it have to be prompted either through a donation, [or] someone noticing it in chat.” Especially in larger streams where there are viewers posting in chat, viewers find it easier to get a streamer’s attention through donations or subscriptions. Two participants in particular recalled specific reactions from other streams. P18 described one stream where a viewer sent a donation with a concerning message about their mental health, to which the streamer stopped the stream and followed up with the viewer:

A donator sent over five bucks [to a streamer] saying, “Hey DrLupo, this is my last five bucks. Enjoy it.” He had ended the stream and hopped on call with this dude. Come back a week later for his next stream and he has an entire mental health-based stream.

As the viewer’s donation message alluded to them planning suicide, the streamer addressed the urgency of the situation in order to provide support to the viewer. The severity of mental health-related messages, as perceived by streamers, may cause them to react differently. While one streamer changed the direction of his stream to address mental health, another streamer simply acknowledged the message of a viewer going through a hard time briefly and continued on with the stream:

They’re really into the game and they’re just focused on trying to win. Then they get a donation or a submessage that says “Hey, I’m going through a rough time right now.” They throw them maybe like $3 or $5. And then … they just kind of brush over it like “Oh, sorry to hear that. Thank you for your money” (P23; male, 21, Hispanic).

P23 explained that this streamer’s channel content focused on competitive gameplay, which may have detracted from the streamer’s ability to respond to donation messages effectively. Depending on the severity of the message, the streamer’s content, and their personality, the two streamers reacted differently to mental health discussions as initiated by donations or subscriptions.

### How Streamers Facilitate Mental Health Discussions (RQ2)

Our second research question focused on how streamers facilitate mental health discussions within their community on gaming streams. Streamers tend to have a direction for their channel and how they want to depict their brand, so they take certain actions to appropriately reflect this idea. We found that streamers generally affected their viewers on the topic of mental health by spreading awareness, connecting with their viewers, and moderating mental health discussions on their channel.

#### Spreading Awareness

Overall, we found that streamers either actively discuss mental health as a main facet of their channel, or moderate the discussion in order to preserve their brand. Some streamers try to educate their viewers or spread awareness about mental health in various forms, including having a therapist on-screen, and talking about mental health articles while gaming. P6 mentioned a time where Twitch streamer Mizkef brought a psychiatrist on stream, explaining how it “seemed like what a normal therapy session would be [asking] questions about how he felt about certain things and what was really bothering him because of the different anxieties … around his success on Twitch.” P17 (male, 28, Caucasian) recalls a time where HealthyGamer_GG (Twitch streamer and psychiatrist) and Reckful started a stream together, and Reckful shared “his experience with depression and struggles with mental health and this guy was kind of coaching them through it or giving some hints, some prompts about how he could work through it.” By sharing their experiences with the community, streamers help lower stigma surrounding mental health. Similarly, viewers like P12 (female, 28, Caucasian) enjoy streaming games and talking about mental health simultaneously to spread awareness.

“I want to play this game and I want to stream and I also want to talk [about] mental health, … okay, here’s some articles I want to talk about and here’s some research that I’ve done” (P12).

In doing so, streamers are able to reach out to viewers on their channel who are there both for the game and the streamer to expose their community to mental health. While some streamers talk about mental health on their channels with psychiatrists or by citing articles, P2 (male, 34, Hispanic) noted that others may host charity streams supporting mental health organizations close to their hearts. These typically occur during game streams, where the streamer will play games for a certain length of time while fundraising, after which all or most of the money accumulated will support that organization. Overall, many participants expressed the importance of making viewers aware of mental health, which is well encapsulated by P19 (male, 21, Caucasian):

“If [streamers are] aware and they show their chat like, ‘Hey, this is a real thing.’ and show that they’re aware of it and are able to bring it up in conversation, I think it’s really powerful. I think it definitely lets everyone know like, ‘Hey, we should be aware of this stuff.”’

#### Connecting With Viewers

Streamers often have a vision for the type of content and atmosphere they want their channel to be and will moderate their channel as such; in some cases, this includes spreading awareness about mental health with the aid of professional resources. In other scenarios, streamers prefer discussing their own experiences with mental health, both while gaming and in Just Chatting streams. Streamers who discuss mental health in Just Chatting streams typically do so to either solely focus on mental health or separate serious conversations from gameplay. P23 explained how some streamers who play more competitive games address mental health conversations in Just Chatting streams separate from their main gameplay. This allows them to focus on their gameplay without answering questions that may be too intensive to address in the midst of a game. Of 25 participants, 17 found that mental health is discussed infrequently during game streams, with a few saying it is more common for streamers to dedicate entire streams to mental health rather than integrate it into their channel. For instance, P18 (male, 18, Caucasian) illustrates how one streamer was able to create a segment on his channel simply dedicated to mental health.

The YouTuber Callmecarson had unique streams on Twitch that specifically were for mental health. after some large-scale drama happen[ed] that allowed for a large audience able to [put aside their feelings] for that conversation and that would probably be the biggest single mental health stream that I’ve seen that has the audience … actually following along (P18).

Having conversations about a personal conflict provides streamers and viewers with the opportunity to discuss difficult issues in their community, strengthening the bond between them. This allows for viewers to feel more connected with the streamer and their community by focusing on the positive aspects of sharing personal experiences rather than the negative.

We found that some of the streamers who dedicated separate streams for mental health rather than talked about it while gaming had either a large viewership, typically over 100k viewers, or played more competitive games. Conversely, streamers who play less competitive games or have a small viewership can afford to speak more about mental health during the game stream. Viewers such as P15 (male, 23, Caucasian) and P24 (non-binary, 28, Caucasian) believe that playing games which are less cognitively demanding (e.g., Stardew Valley) allow streamers to multi-task and hold deeper conversations.

#### Moderating Mental Health Discussions

Whereas some streamers actively discuss mental health during games and just-chatting streams, some streamers prefer to avoid talking about it in fear of hurting their brand and viewership. P15 (male, 23, Caucasian), who has experience with streaming games, explains how the content of his channel is ultimately up to him, and he actively moderates how often he streams and how he goes about responding to his community when discussing mental health. The main reason as to why viewers believe streamers moderate mental health discussions in their gaming streams is that the discussions do not align with the streamer’s goal for their channel and stream. Streamers with the goal of “grabbing viewers” and being active to gain a following usually do not incorporate mental health into their streams, as P19 (male, 21, Caucasian) describes: “I don’t think a lot of people will tie it into whatever they’re streaming about or really make it a point.” Based on what a streamer wants the atmosphere of their stream to be, some participants believe streamers choose to ignore the messages by creating rules for chat, by changing the direction of the conversation to reflect what their goals of the stream are, or by not acknowledging the viewer.

They’ll choose a message and respond, but oftentimes the discussion isn’t continued very often. They want their streams to be in a certain mood and they want conversations to go a certain direction. So I think part of it is that they don’t want to bring their stream down (P7, female, 22, Caucasian).

Usually, especially on smaller channels where often the streamer will respond to you, I think people in their bio usually have a set of rules for chats. So some people say not to bring up sensitive topics like politics or not to ask a streamer certain things cause they otherwise won’t answer (P1, female, 20, Caucasian).

Some participants believe streamers also decide not to engage with toxic and negative commentators as they do not “really pay attention [to] negative stuff. So it’s more so the positive stuff… because he didn’t really feed into what he was being told negatively” (P5, female, 20, Caucasian).

However, streamers may choose to talk more or less about mental health depending on what type of content they are providing to their viewers. Those who are trying to build communities and interact with their viewers are more likely to discuss mental health than those that are just broadcasting gameplay, as illustrated by P11 (male, 19, Caucasian): “The type of streamers who are taking time to talk with your viewers more, not just play the game, probably talk about it more about mental health because mainly streamers just try to focus on their gameplay.” P18 (male, 18 Caucasian) further demonstrates how streamers with the goal of playing games may not be as focused on connecting with viewers during the game stream:

They win tournaments for a living and [then] they divert their focus to talk about, say, mental health. They end up losing a tournament; they lose out on six grand. That’s obviously gonna have an impact on whether or not they’re willing to discuss larger scale questions with their audience. When you do have someone professional going about it and where their focus is dedicated to the game, they’re not going to be more open to talk about it because their focus is located elsewhere.

Especially in competitive gaming streams, where a streamer’s focus needs to be on winning their tournament or game, it may be easier to answer questions from viewers that lack depth rather than ones that require thinking. Because of this, one participant believes that professional gamers with larger followings tend to have a scripted response about heavier topics such as mental health when it is brought up during a game. This allows them to focus on the game stream and briefly address the discussion initiated by viewers without risking losing viewership if they stop playing the game.

### Viewer Reaction to Mental Health in the Gaming Community (RQ3)

Our final research question focused on how viewers reacted to mental health discussions in the gaming community. We found that there were four types of reactions to such discussions: emotional response, offering support, respecting the streamer, and spreading awareness.

#### Emotional Response

Emotional reactions differed in terms of their positivity, but overall, viewers expressed the following: discomfort, joy, sympathy/empathy, and negativity. For some, the level of involvement in the discussion and whether or not they wanted to listen about mental health was dependent on their mood:

I think it depends on what entertainment I’m looking for at the time and what mood I’m in. [If it’s] first person shooter games, in that situation, I don’t want to hear about mental health for various reasons, whether the streamer themselves is bad at talking about it or whether I’m just very interested in the game play at the time” (P23, male, 21, Hispanic).

For other viewers, Twitch is simply not the appropriate place to talk about mental health because streamers are not mental health professionals.

When the guy explained in a heartfelt message that he was depressed to Summit (Summit1G, the streamer), Summit’s actual response to that was “Hey man, don’t be depressed.” And it just honestly made me feel so bad for the guy who was reaching out to him cause he just didn’t know how to respond to him (P23).

When these types of personal problems or situations are brought up, some viewers feel discomfort, since they only planned on watching games. This is prominent in larger streams with more viewers:

“A lot of people just click off because especially when you do have a larger stream with a large personality behind it, people aren’t going to want to learn about specific mental health issues within the context of that game” (P18, male, 18, Caucasian).

For streams more open to mental health discussions, P15 (male, 23, Caucasian) observed how certain mental health topics garnered less interactivity with viewers:

If the topic was like really heavy or if it had something to do with death or suicide, then like people wouldn’t really want to stay for that. But if it has to do with general things like anxiety or OCD, then I’ve noticed people are usually a little bit more chatty about that.

Certain mental health topics carry their own “weight,” and viewers are more likely to feel comfortable talking about lighter topics. Discomfort from these topics does not necessarily come from an emotional response to mental health, but rather from the seriousness of topics like suicide being discussed on an entertainment platform. While “weight” can be a form of stigma, for others comfortability with engaging with mental health topics comes down to emotional capacities and personal boundaries. In essence, viewers seem more likely to feel uncomfortable when mental health is discussed if they believe that Twitch is purely an entertainment platform, if the streamer in question is unable or unwilling to respond appropriately to discussion, or if the topic is particularly stigmatized.

While a few viewers expressed discomfort, others indicated they were glad that it was being talked about. Viewers such as P4 (female, 34, Caucasian) are “happy because they’re talking about it. I don’t think it takes away from the game, from what people are there for.” They believed the exchange was “wholesome”: “I thought it was just wonderful that a streamer could be so kind to their viewers and viewers were so kind to the streamer. It was just such a nice interaction” (P7, female, 22, Caucasian). These viewers are pleased that mental health is being discussed, and, in most cases, do not believe it detracts from the gaming aspect of the stream. On the contrary, they often seem to believe that it helps build the community when paired with the traditional gaming content.

Other viewers echoed this sentiment, sympathizing with what others were going through. In fact, a fifth of our participants felt that they understood what the streamer or viewer was going through, or even had felt it themselves. Those who could sympathize were generally more encouraging toward others sharing their stories as well:

A lot of people start sharing their own stories; when one person starts sharing, other people start sharing. As you’re reading these other stories, you’re seeing these friendships being made because people are going, “Oh my gosh, me too. I understand this.” And it’s really amazing to see people like that come together (P4).

Beyond relating to content creators and fellow viewers who shared personal stories, a fifth of our participants fully empathized with these people. Seeing others in chat talk about mental health caused some viewers to feel they are “not in it alone” (P17, male, 28, Caucasian). This frequently led viewers to be more open about their own experiences and be “able to get through some of what they’re dealing with” (P17). Some streamers play a pivotal role in their viewer’s mental health, and viewers often show them gratitude for it through donations, subscriptions, or writing so in the chat: “You’ll still see instances where people will mention they’re in a bad spot, but the stream is really helping,” to which other viewers respond with comments such as “Oh my God, I feel the same way” and “I’m happy you’re feeling better now” (P19, male, 21, Caucasian).

Although some viewers are motivated to share their personal experiences with mental health, fear of being subject to negativity prevents them from doing so. Viewers such as P4 are upset that some communities within the gaming community are not more supportive of everyone discussing their mental health. To resolve this, many streamers have heavily moderated chats that remove negative messages from trolls to keep the atmosphere more positive:

I think the community and overall moderation is pretty well … [if] someone were to negatively react, if someone were to rag on someone for discussing their mental health struggles … they’re typically kind of banned or have their messages deleted pretty quickly (P17).

We found that moderated chats tend to reflect strong community norms, where many veteran members know how to appropriately act in the chat. Those who do not get their messages deleted, or banned by the moderators.

Despite this range of possible emotional reactions, three participants indicated they felt no emotional response to discussions about mental health. They “feel very neutral” (P11, male, 19, Caucasian). They were indifferent to all conversations surrounding mental health and did not feel as though it affected them personally. One participant recalled several times where a streamer cried on stream, but did not receive an emotional response:

One viewer [would go] to another stream and [say], “Oh this person is crying. Oh this person is whatever.” All those viewers will hop onto that stream just to see what’s going on. But it wouldn’t be with actual concern. It would just be “What’s happening? What’s the drama?” (P10).

People from other streams visited this streamer’s channel in order to know what was happening. As these viewers were only interested in the potential drama surrounding a streamer crying, they left as soon as she felt better and showed no emotional response toward the streamer.

Viewers who watch gaming streamers primarily for their personality tend to be more tolerant of mental health conversations, even if they otherwise feel indifferent:

I view it as kind of a natural progression because [when] someone’s playing a game that’s associated with being easy or you’re supposed to play with friends. You’re watching it for the personality of the people who are playing the game, not for the individual game play or the high skill levels of game play and you have to accept everything that comes with that (P18).

These viewers will accept that there will be some conversation topics, such as mental health, that do not interest them when watching a streamer for their personality. This applies to situations where streamers briefly take a “mental health break” in between games for stress relief. Viewers such as P17 may not be interested in those conversations but realize it comes with the content they enjoy. While five participants described indifference or discomfort, 20 participants expressed positive reactions such as joy, acceptance, sympathy, and empathy toward mental health discussions in gaming streams.

#### Offering Support

Based on the emotional response participants felt, they would offer support to streamers and community members who discussed their experiences with mental health. This support most often comes in the form of emotional support, but can also consist of suggesting activities and personally reaching out to streamers. Some viewers offer emotional support to the streamer by trying to “pick them up to try and be very supportive of them” (P19, male, 21, Caucasian). Just like sympathizers and empathizers, some offered their support in the form of advice, such as: “find or stay with therapy,” “stay active,” and talk “to people they care about” (P24, non-binary, 28, Caucasian). Viewers provide supporting and encouraging comments to show the streamer they are willing to listen. Others described that in reciprocation to the streamer’s and community’s stories, viewers will share their own experiences to “learn and listen and maybe contribute to a conversation” (P15, male, 23, Caucasian). P11 (male, 19, Caucasian) recounts one impactful instance in which a streamer was on a voice call with another streamer when they began talking about an emotional mental health experience:

He started to hyperventilate a bit, which got us worried… Everyone tried to calm him down because he kind of started to cry. And they were trying to calm him down and he was talking with one friend on the voice program Discord. And he started [trying] to calm him down.

While many viewers provide support, personal experience dictates how competently viewers feel they are able to do so. P6 (female, 24, Hispanic), when asked how she reacts to mental health conversations on stream, says “I know what it’s like to be in that situation. If I can, I’ll be helpful. But I feel like I’m. super bad at stuff like that.” Without prior experience with a similar situation to one being discussed, viewers such as P6 are less likely to voice support for someone who discusses mental health issues.

The support of those who are closer to the streamer, as a result, tends to have a greater effect. For instance, P12 (female, 28, Caucasian) feels more comfortable reaching out to streamers she knows personally outside of their stream. She prefers to reach out to the streamer individually, as she did in one instance where the streamer mentioned he was anxious due to life events: “I’m going to DM this person and say, ‘Hey, you’re not alone, feeling anxious. I’m wishing you the best. Let me know if there’s anything you need me to do.”’ This participant, who has experience streaming herself, even offered to cover her friend’s stream so they could “take a break and focus on real life stuff” (P12). Viewers such as these believe that streaming comes secondary to situations happening in “real-life,” such as dealing with one’s own mental health. We found that viewers close to or who have interacted with the streamer before will take the extra step from giving support on stream and reach out to them privately to offer tangible support. This is evident in relationships such as the one between P2 and the streamer he moderates for:

She calls me her rock sometimes. She’s very trusting of me. If I need to take action on something, she’ll trust me for what I need to do. She has just been forthright in saying, “You know, sometimes I disappear because I’m having a depressive episode.”

The established relationship between a streamer and a moderator allows moderators to be more likely to offer direct support to their streamers. Similarly, streamers are more likely to see their moderators as a support system when compared to viewers they do not have a personal relationship with.

#### Respect for Streamers

One of the most common responses our participants had for streamers who discuss mental health is an increase in respect for the streamer. Most of these reactions stem from the belief in the importance in publicly talking about mental health to alleviate stigma, as well as the display of authenticity by the streamer. Many of these viewers “appreciate the transparency” and “that they open up to [viewers]” (P2, male, 34, Hispanic; P10, female, 23, Hispanic). Viewers like these understand that it is difficult to talk about one’s own mental health, and appreciate that streamers wish to bond with their community by discussing topics like these.

I’m proud of them because it takes a lot to be able to talk about mental health, whether it’s their own personal stories or whether it’s just a general talk about hatred, being bullied, … these things that kind of eat at people, I’m happy to see that they take the time to open up about themselves … or want to be more engaging in that specific avenue with their community (P3, male, 36, Caucasian).

These viewers are often watching for the streamer, as opposed to being entertained by the stream’s content: “There’s viewers that are just in there to watch the game play and there’s viewers that are there for the streamer. I am more in it for the streamer that’s streaming the game play. So I appreciate it” (P10). In some viewers, this respect translates into a higher degree of focus dedicated to the stream when these kinds of conversations are happening: “I’ve just turned off [my] game and watched them and paid attention” (P20, male, 22, Hispanic). As mentioned earlier, some streamers prefer to separate their game play from discussions, and others prefer integrating the two.

#### Spreading Awareness

Lastly, some participants demonstrated support for mental health discussions as they understand how it affects societal beliefs. About a quarter of participants believed that talking more about mental health and teaching viewers about it leads to stigma reduction by normalizing the conversations. One way streamers increase awareness about mental health is through fundraising streams. They dedicate a gaming stream to a mental health charity/organization, which tends to receive a lot of attention from viewers (P3, male, 36, Caucasian). Although this is more of the exception than the rule, when done, our participants report that streamers often have a lot of success in raising large sums of money for mental health causes in this way (P18; male, 18 Caucasian).

However, as P22 (female, 29, Caucasian, streamer) explains, because some viewers, especially younger ones, are attention-seeking, when they see people with real issues talking to streamers, they do the same in the hopes of getting a response.

In these cases where a streamer is unsure of the intent behind a message, they may use it as a teaching moment to spread awareness by discussing their own experiences or knowledge about mental health on their channel. Viewers such as P5 (female, 20, Caucasian) believe that mental health is “a good conversation to have” because, although there are many components to mental health, making people more aware of it will make it easier to talk about it in subsequent conversations. Creating an open space for stigmatized topics like mental health demonstrates to viewers that it is acceptable to talk about with others without being judged. Some participants believe that mental health conversations should be as normal as conversations about physical health, as described by P6 (female, 24, Hispanic):

“It makes it more like a normal thing in our lives that we don’t try and shy away from. Cause it’s just like, ‘Oh no, we don’t talk about depression. What will somebody else think?’ It’s just like, ‘Well, what would somebody else think if you had a cough?”’

Exposing viewers to more mental health discussions within the gaming community reduces not only public stigma, but also self-stigma. According to P1 (female, 20, Caucasian), viewers can reduce self-stigma by listening to streamers who discuss mental health:

[When streamers] talk about mental health in a way to destigmatize it kind of makes you feel like anything that you’re dealing with is more okay…. It’s not that people judge others for having mental health issues. It’s necessarily that people often judge themselves for having mental health issues or don’t want to come to terms with it.

Viewers who wish to normalize talking about mental health appreciate when streamers bring it up both during game streams and otherwise, as opening more avenues of communication de-stigmatizes having mental health conversations.

As streamers continue to spread awareness about mental health, viewers are more inclined to think twice about how they respond to conversations about mental health. More viewers are becoming aware of the effect their words can have on others, especially because the intent of a message can be perceived differently than what was intended.

If [gaming] was their one escape and … their family life is just plummeting and they’re like. “Dude, I can’t beat this level. I’m a garbage being.” And when you have that within the context of say another person who’s engaging in self-deprecating humor saying, “Hey, oh dude, man, I’m just so utter shit at this game, I can’t even beat this level.” Like, you can’t tell the difference in intention there (P18).

As P18 describes, in a world of self-deprecating humor and sarcasm, every comment has to be taken with a grain of salt, as it is impossible to understand the intent behind a message without context. For this reason, more viewers are careful about how they respond to comments. Viewers such as P3 believe people should be able to discuss mental health “without fear of reprisal or somebody retaliating or being upset that, ‘how dare you talk about it,”’ and stress the importance of having a welcoming space where people can talk about anything.

## Discussion

Our results show for our participants that the way mental health discussions occur on Twitch (RQ1) depends largely on how individual streamers build their communities. While we do not know how much influencers affect public health, celebrities have shown influence on people’s health behaviors (Hoffman and Tan, 2015). In the gaming community, streamers are able to influence their culture around mental health not only through creating channel rules or assigning a moderating team, but in their choices regarding if and how they discuss mental health. A notable example of this is our finding that streamers who played relaxed games (e.g., Stardew Valley) were more receptive to mental health conversations than those that played more competitive games (e.g., League of Legends). Streamers who play competitive games and want to discuss mental health may have to do so at the potential detriment of both their gameplay and the on-camera persona that they have carefully developed. From the viewer’s perspective, many streamers opt to respond to conversations inadequately (e.g., brushing off a declaration of suicidal intent with an inane “feel better”), use a scripted response to all mental health concerns among viewers as they arise, or avoid the topic to the best of their ability. That said, those who do want to discuss mental health in a constructive way, incorporate it as a segment in their entertainment schedule, such as inviting guest speakers or hosting a Just Chatting stream after their gameplay. Viewers also reported that, in some cases, the streamer’s high skill level allowed them to multitask talking to their viewers and playing the game, and so mental health discussions could be more spontaneous on those streams, irrespective of the game being played. Depending on how the streamer has built their community, discussions will arise differently (e.g., streamer-initiated, chat-initiated, and donation-based), and will resolve according to the individual stream’s culture (e.g., comforting the victim, having open discussion, and trolling in chat).

While results from RQ1 partially answer RQ2 (how do streamers facilitate discussions about mental health in their community?), we found additional nuances when we looked closer at the streamers our participants discussed. More specifically, we observed that in our sample, streamers’ mental health discussion choices (or lack thereof) are often related to their self-presentation and branding. In other words, while there are streamers who voice whatever they want on their platforms without a care, as it is theirs to present in whichever way they choose, other streamers are more concerned with consistency with their brand. Some channels brought up by participants are mental health-focused; however, the majority of the streams mentioned by our participants focused primarily on gaming. While a streamer who is gaming-, comedy-, or “positive vibes-” focused may go through mental health struggles and want to touch upon them, it may not seem consistent with their brand to do so regularly, as suggested by the results of Hamilton et al. (2014) and Chen and Lin (2018). These streamers may not talk about mental health in fear of losing viewers and, consequently, their source of income. Streamers are concerned with viewership and what their audience tunes in for, as discussed in RQ1: streamers know their community’s culture because they built it themselves. This also reveals a potential conflict for streamers who are both monetization- and authenticity-focused, as who they genuinely are may not sell as well as who they portray on camera. However, since this study was conducted from the perspective of viewers, future research may want to further explore this concept from the streamers’ perspectives.

For RQ3 (“What is the viewer reaction to mental health discussions in gaming live streams?”), we found several key factors that seem to influence viewer reactions from our participants, the first of which is their motivation to watch the stream in question. Viewers who watch streams for gameplay-related reasons, such as to learn about strategies used by other players, or just to be entertained, may respond differently to these conversations than those who watch to be involved with a streamer’s community (see Hamilton et al., 2014; Hilvert-Bruce et al., 2018). Additionally, viewers who watch streams for the personality of the streamer may also react differently to conversations on mental health from those whose primary motivation is the gameplay on stream (Deng et al., 2015). This creates a conflict in viewers between the perceived culture of the platform (Twitch) and the perceived culture of individual channels. While many of our participants who were motivated by learning or a desire for entertainment felt that Twitch was not the right place to talk about mental health because it was a place to go and be entertained (platform culture), many other participants who prioritized individual streamers’ communities mentioned full channels dedicated to discussing mental health, in addition to other channels who prioritize mental health despite being mostly dedicated to gaming (individual channel culture). Furthermore, discussing mental health (as long as no self-destructive behavior, harassment, violence, or threats arise) does not violate Twitch’s terms of service, so the platform itself is not necessarily barring the topic. In fact, Twitch has taken it upon themselves to harness the power of their community to reduce the stigmatization of mental health (Twitch Blog, 2018). This difference between platform and channel culture may be related to the interactive nature of live streaming, which leads to communities forming that are centered around the streamer. This means that, in spite of whatever marketing Twitch wants to sponsor, streamers have the opportunity to ultimately decide whether or not their channel is the “right place” for mental health discussions, from the perspective of our viewers. Viewers who find themselves at odds with the streamer’s choices could either leave the stream to find a stream that satisfies their desires or express their distaste in chat.

Many viewers had different opinions on whether Twitch is the “right” place for serious discussions or not; our results showed examples of how an audience may be receptive to the topic, but simply not on Twitch. Previous studies indicate entertainment and knowledge of gaming to be the most common motivation to watching Twitch streams (Hamilton et al., 2014; Chen and Lin, 2018). As such, gaming streamers are expected to almost always be gaming, therefore streamers may be under pressure to provide more entertainment rather than discuss serious topics. Participants noted how some streamers reserved serious discussions for specifically off-stream, their other platforms such as Discord, Twitter, and YouTube. Twitch is about live entertainment and no matter how genuine a streamer’s persona is, streaming is still a raw, unedited performance where streamers must be conscious of their every word and action. Because of this, it is possibly easier to delve into serious topics on these other platforms because they are not live.

Additionally, some participants mentioned that it is difficult to determine the intent behind a message, whether that is one asking for support, or the one giving support during mental health discussions. Though participants label some viewers as “attention-seeking” and trolls in our analyses, this only represents the opinions of these viewers; it is possible that the commenters were, in fact, genuine, but we cannot be certain, as we only have access to the bystanders’ perspectives. This is almost certainly due to the affordances and limitations of textual computer-mediated communication in the case of viewers questioning the motives of fellow viewers (see Fox and McEwan, 2017). It also remains unclear if the reason viewers and streamers suggest resources to those talking about their mental health stems from wanting to support those struggling, or to limit discussion on mental health. It is possible that people genuinely want to support others and get them help, and believe that giving suffering people access to resources like counseling or information pages is the best way available to them. However, it could be that they want to “get rid of the problem,” so the discussion stops and the game-stream can continue uninterrupted. The question of authenticity remains an important one in the minds of viewers, and should be considered carefully when examining live streaming communities, gaming-focused or otherwise.

Finally, as streamers spread awareness about mental health during and separate from their gameplay, some viewers are more careful of how they choose to respond to comments surrounding mental health, which is also demonstrated in Lee et al. (2020). They are more understanding of how their words will be perceived to others and ensure their message is not wrongly received. This leads to greater understanding toward people with mental health issues, as some viewers will be more understanding outside of Twitch to those with a mental health illness. With the increase of and push to normalize spreading awareness of mental health, existing negativity surrounding the issue may start to decline, allowing an overall decrease in stigma on the platform (Vidourek and Burbage, 2019); however, further research would be needed to see its effect in full. It also points out avenues for interventions when it comes to mental health crises on social media platforms. By harnessing the power of micro-celebrities, particularly within niche communities like competitive fighting games or mukbangs, people who are “immune” to the traditional advertising techniques employed by public health campaigns can be reached and their behaviors changed.

### Limitations and Future Directions

Although this study has broken new ground in terms of understanding the relationship between mental health and gaming in live streaming communities, it is not without its limitations. For one, this study primarily focused on Twitch in terms of our recruitment and analysis, although many other streaming platforms, such as YouTube Gaming, exist. Another limitation is the fact that this study was a small-scale qualitative piece, so we could not find as many cross-genre patterns as a large-scale study might have. A larger study may be able to find various patterns between different genres of games as it would yield more results about mental health discussions in each community. Further, we discuss how viewers may make pleas to get the streamer’s attention (see section “Cries for Help”). Our data do not explore how streamers and moderators judge how a real cry for help differs from a fake one. There may be implications to misjudging these cries; however, further research is needed to understand how streamers and moderators come to such conclusions. Additionally, our sample size mostly consisted of Caucasian, English-speaking participants from the United States, with a few exceptions. Our sample consisted of people who knew the topic of the study was mental health and wanted to discuss it, signifying some self-selection bias. As such, it is important to note our results cannot be generalized to a large viewer population who do not have a predisposition to discuss mental health. These results may also not apply to viewers who are international, are apathetic toward mental health discussions, and have an opinion on it but have not experienced it.

Future research could explore many possible avenues within mental health in the gaming community. First, scholars could take a closer look at how gamers benefit from participating in live stream communities, such as gaining social capital or learning coping strategies. As this research focused on viewer perceptions of mental health, researchers may also want to explore streamers’ perspectives on such discussions, specifically in regard of authenticity and monetization. Another study could also examine cross-platform and cross-genre differences with a larger-scale study. During our interviews, we noticed participants occasionally bringing up other social media platforms such as Twitter. It would also be valuable to consider the norms of mental health and gaming discussions on those platforms. As discussed before, we found that streamers moderate their channels to reflect their brand. By delving into how streamers moderate their viewers’ involvement surrounding mental health discussions, researchers could complete the partial image of mental health and gaming that we have illustrated in the present study.

## Conclusion

In conclusion, the present study has highlighted Twitch as a popular platform for mental health discussions, both between streamers and their viewers, as well as between fellow viewers. These can be as formal as scheduled sessions the streamer sets aside because of their own passion for the topic, or as informal as one viewer calling out for help and others in the chat typing to their rescue. Despite this multitude of conversations, conflicts still arise. Viewers who tune into Twitch.tv for entertainment face a choice: stay to support either the viewer in crisis or the streamer, or leave to seek other sources of entertainment. This decision is complicated by the fact that Twitch viewers cannot determine the legitimacy of either the streamer or their fellow viewers’ cries for help, and are sometimes unsure of whether interacting with a channel is making a real difference for the person in question. All that said, Twitch.tv is still an important place for communities to gather, the gaming community in particular, and is a source of practical resources and simple belonging for many. Though imperfect, Twitch.tv lets people relate to one another, and for many gamers, this includes a side of mental health with their favorite games.

## Data Availability Statement

The raw data supporting the conclusions of this article will be made available by the authors, without undue reservation.

## Ethics Statement

The studies involving human participants were reviewed and approved by the Institutional Review Board of the New Jersey Institute of Technology. The patients/participants provided their written informed consent to participate in this study. Written informed consent was obtained from the individual(s) for the publication of any potentially identifiable images or data included in this article.

## Author Contributions

RG designed the interview protocol (along with NL), wrote the bulk of the introduction and literature review, as well as parts of the results and discussion, and served as the project manager. CC wrote the rest of the introduction and literature review, as well as parts of the results and discussion, and was in charge of putting together the last revisions and creating all submission documents. NL and JU wrote the methodology section, as well as parts of the results and discussion sections. DW was the project supervisor and provided critical feedback throughout the entire writing process. All authors conducted interviews and participated in the data analysis.

## Conflict of Interest

The authors declare that the research was conducted in the absence of any commercial or financial relationships that could be construed as a potential conflict of interest.

## Footnotes

1 TwitchCon is an annual convention for Twitch enthusiasts and content creators.

2 Moderators are viewers who take on additional roles in facilitating a live stream in accordance to streamers’ guidelines (Wohn, 2019).

3 Steam is a digital content distribution service.

4 Destiny is a multiplayer first-person shooter video game.
