# Supplementary material for: An Exploration of Mental Health Discussions in Live Streaming Gaming Communities
Source: Front Psychol. 2021 Mar 16;12:575653. doi: 10.3389/fpsyg.2021.575653 (PMC8007960; doi:10.3389/fpsyg.2021.575653)
Supplement: Supplementary file 1 [file Data_Sheet_1.PDF]

Twitch and Mental Health

Interview Protocol

Hi, my name is [name] and I am a research assistant at NJIT's Social X Lab. You agreed to participate in a study concerning your views on mental health on Twitch. For this interview, I'd like to hear about your thoughts concerning your own experiences with mental health conversations on Twitch, your favorite streamers who talk about the topic, and your relationship with mental health. As this can be a sensitive subject, you can ask me to skip any questions that seem too personal or otherwise make you feel uncomfortable answering.

Background questions

Whenever you feel ready, I'll ask you a few questions about the content you watch on Twitch and how you watch it.

1. How often do you watch channels on Twitch on a weekly basis?
2. How often do you stream on Twitch on a weekly basis?
    a. What kind of content do you stream on Twitch?
3. What genres of streamers do you watch?
4. Who are some of the streamers that you watch?
5. At what time of the day do you usually watch Twitch streams?

Impressions of Mental Health

Now that I have a sense of how often you're on Twitch and the types of communities you're a part of, let's talk about mental health conversations on Twitch.

1. How often do streamers discuss mental health?
    a. Are they mental health professionals or mental health advocates?
2. How often would you say that mental health is discussed in Twitch streams?
    a. Who initiates these discussions?
3. How often would you say streamers talk about mental health outside of Twitch?
4. Do you actively follow streamers who often discuss mental health topics?
5. How do you react when you hear streamers talk about mental health topics?
6. How do other people react in chat when they hear streamers talk about mental health topics?
7. Have you seen someone initiate a conversation about mental health in a stream's chat?
    a. How did you react to that?
    b. How did the streamer react to that?
    c. How do you think a streamer should react to chat messages about mental health?

8. When, if ever, is it appropriate to talk about mental health topics during streams?

Streamer Profile

Think about one of your favorite gaming streamers who have talked about mental illness in the past.

1. What is the name of their channel?
    a. What are their age and gender?
    b. Do you know where they are located?
    c. What is their race/ethnicity?
    d. Do you know what their sexual orientation is?
        i. Do they identify as transgender?
    e. What games do they usually play?
    f. What is the size of the stream?
2. Is mental health the main focus of your streamer's channel?
3. Are they a mental health professional?
4. Do they currently or in the past have a disclosed mental illness?
5. Do they talk about their own experiences or someone else's experiences?

Relationship to Streamer

1. When did you start watching the streamer's channel?
2. What caused you to start watching that channel?
3. How close do you feel towards the streamer and their community?
    a. [If interviewee feels close to streamer] How did conversations about mental health contribute towards this closeness?

Streamer and Mental Health

1. How often does this streamer discuss mental health?
2. What kinds of mental health disorders did the streamer talk about?
    - (ie. Anxiety, Depression, Personality Disorders, Eating Disorders, others)
    - What were their experiences with [said mental health disorder]?
3. Was the streamer talking about their personal experiences with mental health?
    - If so, did the conversation change your opinion on the streamer?
    - If not their personal experiences, what did the streamer discuss?
4. How did you respond to the streamer as they were talking about mental health?
    - Did you respond in any way outside of the streamer's Twitch chat?
    - Did you provide any type of emotional support when the streamer talked about their mental health history?
5. Have you left the channel if streamers began to discuss mental health?
    - If so, why did you leave the channel?

        - How many other viewers did you see leave the channel if streamers began to discuss mental health?
6. Describe what kinds of replies you have seen from other viewers while the streamer talked about mental health.

Gaming and Mental Health

1. What is the streamer doing while they talk about mental health?
    a. Do they play these games while or in between discussing mental health topics?
2. Does the discussion affect the game-play or vice versa?
3. How does the gaming community's reaction impact their mental health?
    a. For example: the gaming community at large, the community of their specific game(s), and the community of their stream

    Now I'm going to ask you questions not specific to a streamer.

4. What is the culture around streamers playing games and discussing mental health?
    a. How do the viewers react to it?
5. Is it common for most gamers to talk about mental health while they are gaming?
6. How do you feel when streamers talk about mental health during the game stream?

Education and Charity

1. Have streamers ever encouraged you to donate to mental health causes?
    a. If so, which ones?
2. Have you donated to mental health causes because of streamers?
    a. If so, what did the streamer say that made you want to donate?
3. Did you learn more about mental health from the streamer?
    a. Do you think other viewers learned from the streamer?

Opinions on Mental Health

I'm now going to ask questions not directly related to Twitch, but rather mental health in general.

1. How do you react when hearing others talk about mental health?
2. Who do you think is qualified to speak about mental health?
    a. What gives someone those qualifications?
3. What do you think differentiates a mental health professional and a mental health advocate?
4. What is an example of a good time to discuss mental health?
5. Do you feel it is necessary to have public discussions about mental health? Why?

Personal Questions

Would you be okay discussing your personal mental health? You do not have to answer these questions if you choose not to.

(If no, skip to personal demographics.)

1. How often, in a non-professional context, do you talk about mental illness?
    a. Is this done more online or offline?
2. How often, in a non-professional context, does someone else talk to you about mental illness?
3. Do you have a history of mental health disorders?
    a. Are you comfortable with telling me about your experience with your disorder(s)?
        i. (If yes,) How would you say that your experience with mental health disorders has impacted your relationship with them?

Thank you for sharing your experiences.

4. Do you know someone close to you who is experiencing a mental health disorder?
5. Is there a feature Twitch could add to help facilitate mental health discussions?

Personal Demographics

1. What is your age?
2. What is your gender?
3. What is your ethnicity or racial identity? (If they answer nationality, follow up with "Alright, with which ethnicity or race do you identify?")
